# Critical Points in the Noiseberg Achievable Region of the Gaussian Z-Interference Channel

**DOI:** 10.3390/e26110898

**Published:** 2024-10-23

**Authors:** Max H. M. Costa, Chandra Nair, David Ng

**Affiliations:** 1 School of Electrical and Computer Engineering, Universidade Estadual de Campinas (Unicamp), Campinas 13083-852, SP, Brazil; 2 Department of Information Engineering, The Chinese University of Hong Kong, Sha Tin, Hong Kong; cnair@ie.cuhk.edu.hk (C.N.); davngz@gmail.com (D.N.)

**Keywords:** shannon theory, interference channels, gaussian interference, noiseberg multiplexing

## Abstract

The Gaussian signaling strategy with power control for the Gaussian Z-interference channel with weak interference is reviewed in this paper. In particular, we study the various communication strategies that may arise at various points of the capacity region and identify the locations of the phase transitions between the various strategies. The Gaussian Z-interference channel with weak interference is known to have two critical points in its capacity region, where the slope of the region shows a sudden change. They occur at the points of the unconditional maximum rate for one of the users and the maximum rate that can be accommodated by the other user. In this paper, we discuss additional critical points (locations of phase transitions) in the achievable region of this channel. These turn out to be second-order phase transitions, i.e., we do not observe a discontinuous slope in the achievable rate region, but there is a discontinuity in the second derivative of the rate contour of the achievable region. This review paper is mainly based on some of our ITA (Information Theory and Applications Workshop, UCSD, San Diego, CA, USA) papers since 2011.

## 1. Introduction

Many authors have studied scalar Gaussian interference channels since 1974 [1,2,3,4,5,6,7,8,9,10,11,12,13,14,15,16,17,18,19,20,21,22,23]. One of the key questions in this area, for which we do not have a definitive answer as yet, is whether Han–Kobayashi inner bound with Gaussian signaling achieves the capacity region.

**Figure 1:**
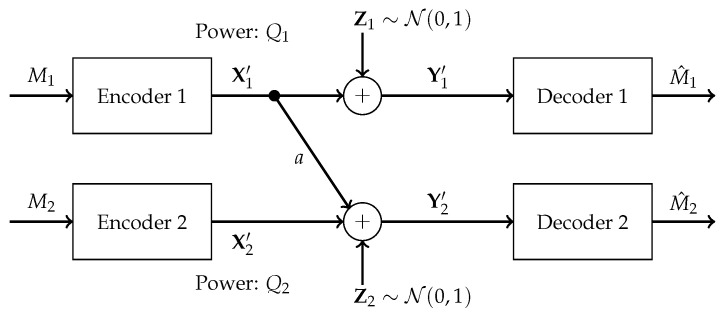
Gaussian *Z*-interference channel.

The model under investigation in this paper is the one-sided interference channel given by $Y_{1}^{\prime }=X_{1}^{\prime }+Z_{1}^{\prime }$ and $Y_{2}^{\prime }=X_{2}^{\prime }+aX_{1}^{\prime }+Z_{2}^{\prime }$, where $X_{1}^{\prime }$ and $X_{2}^{\prime }$ are transmitter signals constrained to have average powers $Q_{1}$ and $Q_{2}$, respectively, *a* is an interference gain in the interval (0,1), $Z_{1}^{\prime }$ and $Z_{2}^{\prime }$ are Gaussian noises of unit variance, and $Y_{1}^{\prime }$ and $Y_{2}^{\prime }$ are the two received signals. This model is depicted in Figure 1.

**Figure 2:**
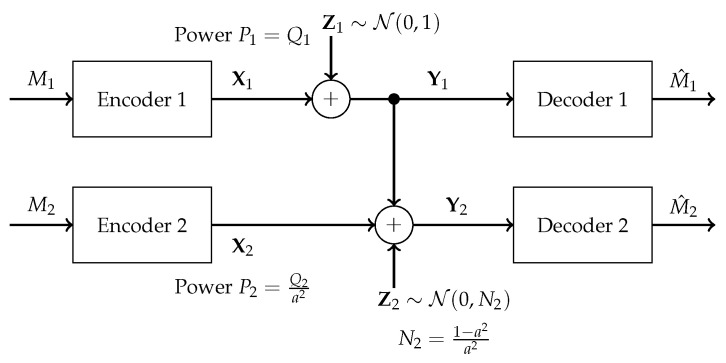
Degraded Gaussian interference channel.

The receivers are interested in messages sent by their respective, same-indexed, transmitters. Thus $X_{1}^{\prime }$ encodes a message addressed to receiver 1 and $X_{2}^{\prime }$ conveys a message to receiver 2. This model is a particular case of the Gaussian interference channel, which exhibits interference in both directions. Like in the more general model, the problem of finding the associated capacity region has been open for almost 50 years. In the case of strong interference, when a≥1, the capacity region is known [6,7]. In this case, the unintended receiver can fully decode the interfering message. Therefore, the rate region coincides with the intersection of the two underlying multiple access channel regions. Also, when a=0, the problem has a trivial solution. This paper uses the fact that the Gaussian Z-interference channel with interference parameter *a* in the range (0,1) can be regarded as a degraded Gaussian interference channel [8], a model shown in Figure 2.

Like the Gaussian Z-interference channel, the degraded Gaussian interference channel is characterized by three parameters: The two powers $P_{1}$ and $P_{2}$, and the additional independent noise in the second receiver, power $N_{2}$. These parameters are related to the parameters of the original Z-interference channel by $P_{1}=Q_{1}$, $P_{2}=Q_{2}/a^{2}$ and $N_{2}=\left(1-a^{2}\right)/a^{2}$. Moreover, since 0<a<1, the additional noise power $N_{2}$ is always positive. For simplicity, we choose to use the more common notation, without the primes, in this channel, which will constitute our working model.

In this review paper, we investigate the behavior of the noiseberg encoding scheme [16], which has recently been shown [24] to coincide with the Han–Kobayashi region with Gaussian signaling. From a communication engineer’s perspective, knowing the optimal Gaussian signaling strategy for a given set of parameters is essential when maximizing $R_{1}+\beta R_{2}$. In particular, we investigate additional critical points in the achievable region of the noiseberg scheme for the Gaussian Z-interference channel with weak interference. These critical points are associated with transitions between different modes of operation. A third critical point happens between the so-called multiplex and the overflow regions produced in the noiseberg encoding scheme [25]. A fourth critical point happens after the overflow mode is in effect, as the evolution of modes leads to a transition to pure superposition or, otherwise, reaches an extreme boundary in the λ × *h* parameter space.

Another possible transition may happen when the noiseberg region takes up all the power available for $P_{1}$ before the noiseberg height reaches the overflow level ($N_{2}$). This will be characterized as a transition from Phase 4 to Phase 7. Subsequently, the optimal path in this case will transition to Phase 6 and, finally, to Phase 3.

## 2. Preliminaries

**Figure 3:**
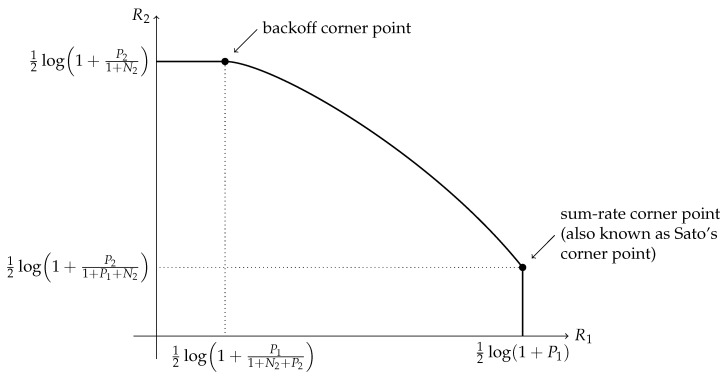
Critical points of the capacity region.

We focus on the degraded interference channel model depicted in Figure 2. Two extreme points in the channel capacity region have been identified for this channel. One extreme point occurs when $X_{1}$ sends information at its maximum possible rate and $X_{2}$ uses what is left of the channel, with $X_{1}$’s interference treated as noise. In this extreme point the achieved rate pair $\left(R_{1},R_{2}\right)$ is given by $R_{1}=\frac{1}{2}\log\left(1+P_{1}\right)$ and $R_{2}=\frac{1}{2}\log\left(1+\frac{P_{2}}{1+P_{1}+N_{2}}\right)$ (cf. Figure 3). There is a slope discontinuity for the capacity region at this extreme point, which follows from the capacity region of an associated degraded broadcast channel [5,8], establishing that this extreme point is a critical point. From this, it immediately follows that this point also maximizes $R_{1}+R_{2}$, the sum-rate, and therefore, this corner point will be referred to as the sum-rate corner point.

Another extreme point in the achievable region occurs when all the privilege of operating at maximum rate is given to the second transmitter [8,26]. In this case, the first transmitter must lower its rate to the point where the second receiver is sure to decode and eliminate all the interference that its signaling might impose. The first transmitter then uses the noisy channel that sees noise power $1+N_{2}+P_{2}$. Therefore, we have $R_{1}=\frac{1}{2}\log\left(1+\frac{P_{1}}{1+N_{2}+P_{2}}\right)$ and $R_{2}=\frac{1}{2}\log\left(1+\frac{P_{2}}{1+N_{2}}\right)$. There is also a slope discontinuity for the capacity region at this extreme point, which follows from a recent outer bound developed in [27]. This corner point is referred to as the backoff corner point.

### 2.1. Noisebergs—A Brief Review

**Figure 4:**
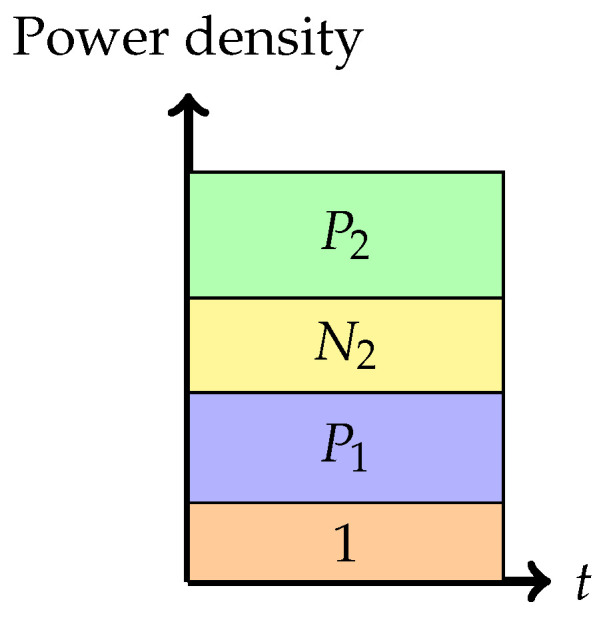
Phase 1 (Sato’s corner point).

**Figure 5:**
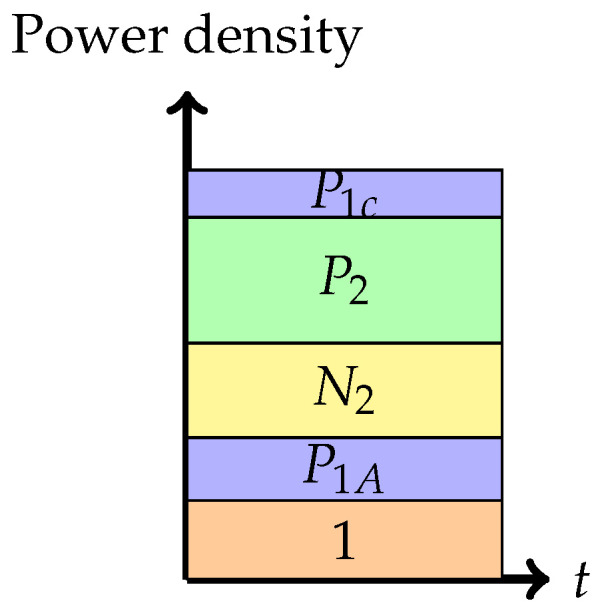
Phase 2 (Pure superposition strategy).

**Figure 6:**
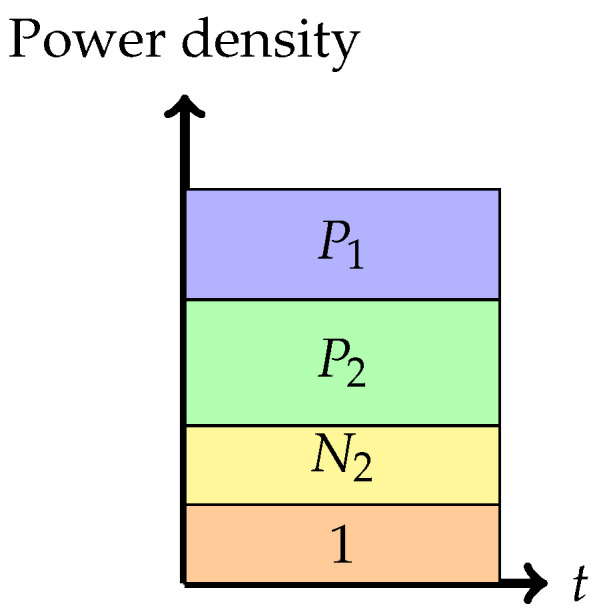
Phase 3 (Backoff corner point).

**Figure 7:**
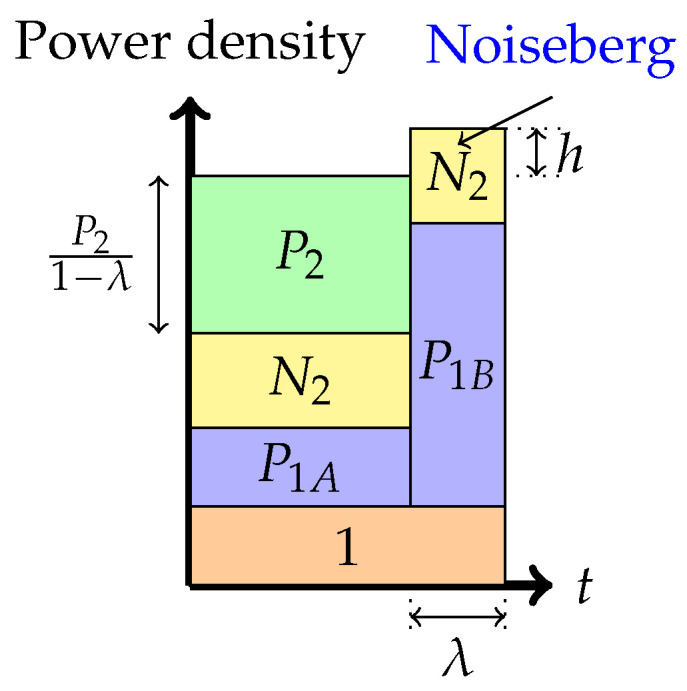
Phase 4 (The noiseberg strategy).

**Figure 8:**
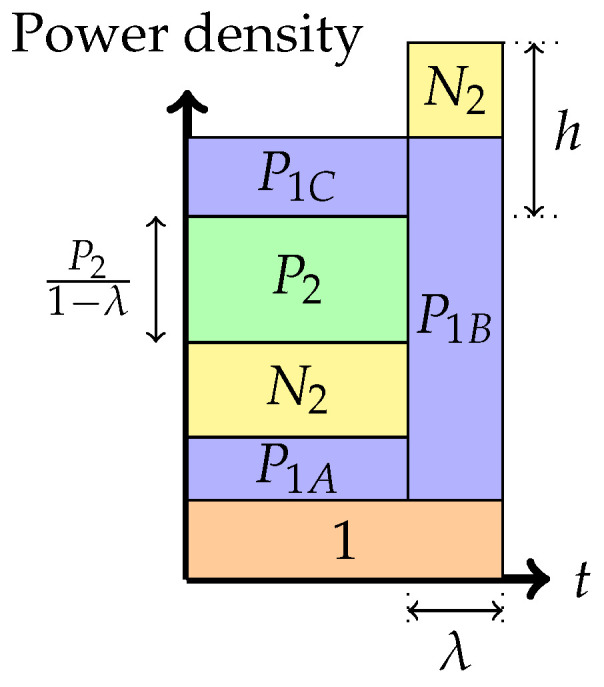
Phase 5 (The overflow region).

**Figure 9:**
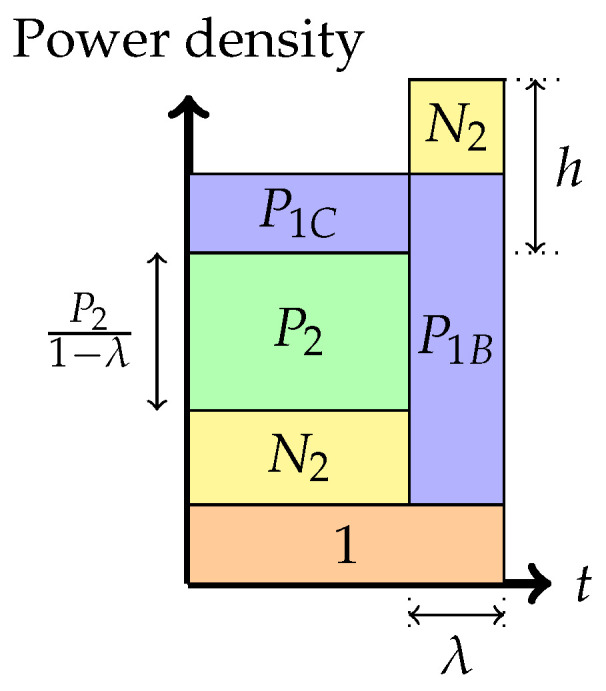
Phase 6 (The top boundary of the admissible (λ,h) parameter region).

**Figure 10:**
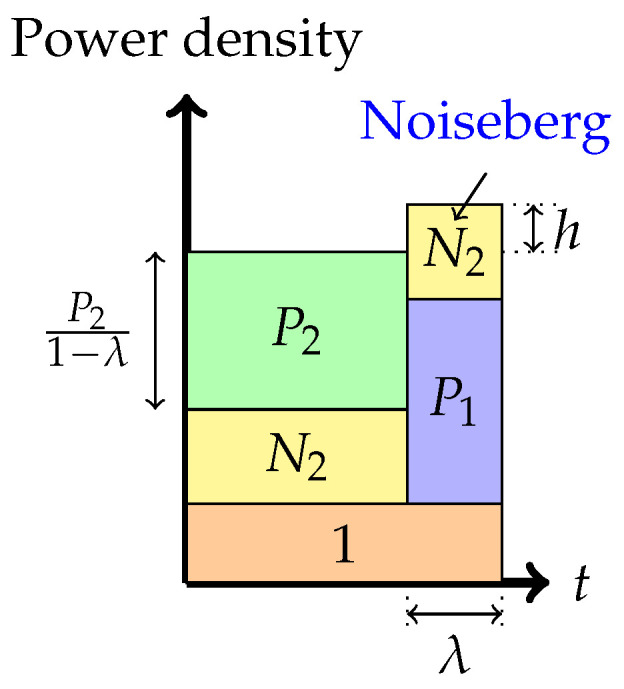
Phase 7 (Essentially, a portion of the so-called non-naïve time sharing).

A noiseberg transmission scheme is a particular Gaussian signaling strategy with power control, with (only) seven potential phases, depicted in Figure 4, Figure 5, Figure 6, Figure 7, Figure 8, Figure 9 and Figure 10. In a nutshell, it is a scheme that combines superposition coding, non-naïve (i.e., power-controlled) time-sharing, and water filling between the two spectral regions that become multiplexed. More specifically, it is a time-sharing/bandwidth-sharing between two signaling strategies, with the first strategy (depicted on the left) occupying (1−λ) fraction of the time (band) and the second strategy occupying the remaining λ fraction of the time (band). In particular, the strategies in Figure 4, Figure 5 and Figure 6 can be considered as special instances of those in Figure 7, Figure 8 and Figure 9, respectively, by setting λ=0. In the leftmost strategy, using band (1−λ), one allocates part of the power budget to combine transmissions to both decoders in a pure superposition manner, exactly as is known to be optimal for Gaussian broadcast channels. In the second strategy, over band λ, the communication is solely accomplished between the first transmitter and the first receiver. Yet, this strategy can also be interpreted as a particular point of the capacity region of a Gaussian broadcast channel, restricted to maximal $R_{1}$ and null $R_{2}$.

Consider the following seven communication strategies, using Gaussian signaling, for the Gaussian interference channel:

1. Phase 1: *Treating interference to be noise at the weaker receiver (Sato’s corner point)*
  In this phase, the weaker receiver, $Y_{2}$, decodes its message by treating $X_{1}$ as noise. This is depicted pictorially in Figure 4. The decoding order in the picture is assumed to go from top to bottom. Any receiver will decode all the messages on top of its message (including its message) in any band by treating those below it as noise. The rate pair achieved in this phase is
  $$\begin{array}{c} R_{1}=\frac{1}{2}\log\left(1+P_{1}\right),R_{2}=\frac{1}{2}\log\left(1+\frac{P_{2}}{1+P_{1}+N_{2}}\right). \end{array}$$

2. Phase 2: *Partial interference cancellation at the weaker receiver (or pure superposition coding)*
  In this phase, the weaker receiver, $Y_{2}$, decodes a part of $X_{1}$ first, subtracts this from the received signal, and then decodes its own signal $X_{2}$. The rate pair achieved in this phase is
  $$\begin{array}{cc} R_{1} & =\frac{1}{2}\log\left(1+\frac{P_{1C}}{1+N_{2}+P_{2}+P_{1A}}\right)+\frac{1}{2}\log\left(1+P_{1A}\right),\\ R_{2} & =\frac{1}{2}\log\left(1+\frac{P_{2}}{1+N_{2}+P_{1A}}\right). \end{array}$$
  Note that $P_{1A}+P_{1C}=P_{1}$. This can be seen as a mix of Phase 1 and Phase 3, to be seen next.

3. Phase 3: *Interference cancellation at the weaker receiver (the backoff corner point)*
  In this phase, the weaker receiver, $Y_{2}$, decodes $X_{1}$ first, subtracts this from the received signal, and finally decodes its own signal $X_{2}$. The rate pair achieved in this phase is
  $$\begin{array}{c} R_{1}=\frac{1}{2}\log\left(1+\frac{P_{1}}{1+N_{2}+P_{2}}\right),R_{2}=\frac{1}{2}\log\left(1+\frac{P_{2}}{1+N_{2}}\right). \end{array}$$
  Note that the rate for message 1 is solely determined by the ability of the weaker decoder to decode it. This encoding strategy is similar to that in a degraded message sets Gaussian broadcast channel where message 1 is meant to be communicated to both receivers.

4. Phase 4: *Time-sharing between the following two strategies: Treating interference to be noise at the weaker receiver and transmitting solely to the stronger receiver (or multiplex strategy)*
  In this phase, there is a time-sharing between two communication schemes. The first scheme, employed for (1−λ) fraction of the time, employs the Phase 1 strategy, and the second scheme consists of transmission only to the stronger receiver. The total average power in each band is indicated in the figure. Therefore, one must divide the power by the band duration to obtain the height. It is this phase that leads to the noiseberg nomenclature. We denote by *h* the height difference between the $N_{2}$ slab in the second band and the power level of $P_{2}$ in the first band. This height difference comes from part of the noise spectrum of $Z_{2}$ that floats above the signal level in the first band and characterizes what we call a noise-iceberg, or *noiseberg*. The flotation of the noise slab releases prime-rate space in the power × bandwidth plane, in a fashion that Archimedes would be sure to appreciate. In this phase, we obtain the following:
  (1) $$\begin{array}{cc} P_{1} & =P_{1A}+P_{1B}\\ \frac{P_{1A}+P_{2}}{1-\lambda } & =\frac{P_{1B}}{\lambda }-h. \end{array}$$
  The rate pairs achieved in this scheme are
  (2) $$\begin{array}{cc} R_{1} & =\frac{\bar{\lambda }}{2}\log\left(1+\frac{\bar{\lambda }P_{1}-\lambda \bar{\lambda }h-\lambda P_{2}}{\bar{\lambda }}\right)+\frac{\lambda }{2}\log\left(1+P_{1}+P_{2}+\bar{\lambda }h\right),\\ R_{2} & =\frac{\bar{\lambda }}{2}\log\left(1+\frac{P_{2}}{\bar{\lambda }\left(1+N_{2}\right)+\bar{\lambda }P_{1}-\lambda \bar{\lambda }h-\lambda P_{2}}\right), \end{array}$$
  where $\bar{\lambda }$ denotes (1−λ).

5. Phase 5: *Time-sharing between the following two strategies: Partial interference cancellation at the weaker receiver and transmitting solely to the stronger receiver (or overflow strategy)*
  In this phase, as before, there is a time-sharing between two communication schemes. The first scheme, employed for (1−λ) fraction of the time, employs the Phase 2 strategy, and the second scheme consists of transmission only to the stronger receiver. The total average power in each band is indicated in the figure. We denote by *h* the height difference between the top of the $N_{2}$ slab in the second band and the power level of $P_{2}$ in the first band. As argued in [16,24], the total heights of the two signal bands must agree via a water-filling argument.
  The noise band of height $N_{2}$ floats above the signal power band in the rightmost strategy. The floating of the noise band frees up prime frequency (or time) space in the spectrum for signal occupation.
  In this phase, we obtain the following:
  (3) $$\begin{array}{cc} P_{1} & =P_{1A}+P_{1B}+P_{1C}\\ \frac{P_{1B}}{\lambda } & =N_{2}+\frac{P_{2}+P_{1A}+P_{1C}}{\bar{\lambda }}\\ \frac{P_{1C}}{1-\lambda } & =h-N_{2} \end{array}$$
  (4) $$\begin{array}{cc} R_{1} & =\frac{\bar{\lambda }}{2}\log\left(1+\frac{\bar{\lambda }P_{1}-\lambda P_{2}-\bar{\lambda }h+{\bar{\lambda }}^{2}N_{2}}{\bar{\lambda }}\right)\\ \; & \;+\frac{\bar{\lambda }}{2}\log\left(1+\frac{\bar{\lambda }\left(h-N_{2}\right)}{\bar{\lambda }\left(1+N_{2}\right)+\bar{\lambda }P_{1}-\lambda P_{2}-\bar{\lambda }h+{\bar{\lambda }}^{2}N_{2}+P_{2}}\right)\\ \; & \;+\frac{\lambda }{2}\log\left(1+P_{1}+P_{2}+\bar{\lambda }N_{2}\right),\\ R_{2} & =\frac{\bar{\lambda }}{2}\log\left(1+\frac{P_{2}}{\bar{\lambda }\left(1+N_{2}\right)+\bar{\lambda }P_{1}-\lambda P_{2}-\bar{\lambda }h+{\bar{\lambda }}^{2}N_{2}}\right). \end{array}$$

6. Phase 6: *Time-sharing between the following two strategies: Interference cancellation at the weaker receiver and transmitting solely to the stronger receiver (top boundary of the admissible parameter region)*
  In this phase, there is also a time-sharing between two communication schemes. The first scheme, employed for (1−λ) fraction of the time, employs the Phase 3 strategy, and the second scheme consists of transmission only to the stronger receiver. The total average power in each band is indicated in the figure. As in Phase 5, we denote by *h* the height difference between the top of the $N_{2}$ slab in the second band and the power level of $P_{2}$ in the first band. Again, as argued in [16,24], the total heights of the two bands must agree via a water-filling argument. In this phase, we obtain the following:
  (5) $$\begin{array}{cc} P_{1} & =P_{1C}+P_{1B}\\ \frac{P_{1B}}{\lambda } & =N_{2}+\frac{P_{2}+P_{1C}}{\bar{\lambda }}\\ \frac{P_{1C}}{1-\lambda } & =h-N_{2} \end{array}$$
  $$\begin{array}{cc} R_{1} & =\frac{\bar{\lambda }}{2}\log\left(1+\frac{\bar{\lambda }P_{1}-\lambda \bar{\lambda }N_{2}-\lambda P_{2}}{\bar{\lambda }\left(1+N_{2}\right)+P_{2}}\right)\\ \; & \;+\frac{\lambda }{2}\log\left(1+P_{1}+P_{2}+\bar{\lambda }N_{2}\right),\\ R_{2} & =\frac{\bar{\lambda }}{2}\log\left(1+\frac{P_{2}}{\bar{\lambda }\left(1+N_{2}\right)}\right). \end{array}$$

7. Phase 7: *Time-division between transmitting solely to the two receivers*
  In this phase, for (1−λ) fraction of the time, we transmit solely to the weaker receiver, and for λ fraction of the time, we transmit solely to the stronger receiver. In this phase, we obtain
  $$\begin{array}{cc} R_{1} & =\frac{\lambda }{2}\log\left(1+\frac{P_{1}}{\lambda }\right),\\ R_{2} & =\frac{\bar{\lambda }}{2}\log\left(1+\frac{P_{2}}{\bar{\lambda }\left(1+N_{2}\right)}\right). \end{array}$$

**Remark 1.**  *A general Gaussian signaling strategy incorporating superposition coding and non-naïve (i.e., power-controlled) time-sharing will have many more phases than those described above. However, it was proposed in [16] and established in [24] that the boundary of the Gaussian signaling region is obtained by restricting observations to these seven strategies*.

### 2.2. The Gaussian Signaling Region

One needs to optimize $R_{1}+\beta R_{2}$ for 1≤β≤∞, among the various signaling strategies or phases to compute the Gaussian signaling region.

#### 2.2.1. Slopes at the Corner Points

It is known [5,8] that $R_{1}+R_{2}$ (i.e., β=1) is maximized (for the capacity region) at the corner point $R_{1}=\frac{1}{2}\log\left(1+P_{1}\right)$ and $R_{2}=\frac{1}{2}\log\left(1+\frac{P_{2}}{1+N_{2}+P_{1}}\right)$ (Note that $R_{1}+\beta R_{2}$ for 0≤β≤1 will also pass through the same corner point, as this corresponds to the maximum value of $R_{1}$). This corresponds to a Phase 1 communication strategy. In particular, it has been shown [28] that the supporting hyperplane, $R_{1}+\beta R_{2}$, will touch the Gaussian signaling region (or equivalently the noiseberg region) at the same corner point if and only if $\beta \leq \beta_{Sato}$ (defined below). Thus, $\beta_{Sato}$ marks the first critical (or phase transition) point of the noiseberg region.

**Theorem 1**  ([28]). *For a Gaussian Z-interference channel, let $\beta_{Sato}$ be the largest value of β≥1 such that*

(6) $$\begin{array}{c} \underset{\left(R_{1},R_{2}\right)\in R^{\mathrm{HK-GS}}}{\sup}\left(R_{1}+\beta R_{2}\right)=\frac{1}{2}\log\left(1+P_{1}\right)+\frac{\lambda }{2}\log\left(1+\frac{P_{2}}{1+N_{2}+P_{1}}\right). \end{array}$$

*Then,*

$$\begin{array}{c} \beta_{\mathrm{Sato}}=\min\left\{\frac{\left(N_{2}+P_{2}\right)\left(1+N_{2}+P_{1}\right)}{P_{2}\left(1+P_{1}\right)},\beta^{*}\right\}, \end{array}$$

where $\beta^{*}$ is the unique positive solution ψ(β)=0, where

$$\begin{array}{cc} \psi (\beta ) & :=\beta \left(\log\left(1+\frac{P_{2}}{1+N_{2}+P_{1}}\right)-\frac{N_{2}P_{2}}{\left(1+N_{2}+P_{1}\right)\left(1+N_{2}+P_{1}+P_{2}\right)}\right)\\ \; & \;+\log\left(1-\frac{P_{2}\left(1+P_{1}\right)}{\left(1+N_{2}+P_{1}\right)\left(1+N_{2}+P_{1}+P_{2}\right)}\beta \right). \end{array}$$

**Remark 2.**  *We do not yet have a matching converse (i.e., one that follows from an outer bound to the capacity region) that establishes the slope at this corner point. An interested reader may refer to [22,27] for recent developments along these lines.*

On the other hand, it is known that for large enough β the supporting hyperplanes to the Gaussian signaling region, [29], as well as the one to the capacity region [27] pass through the backoff corner point established in [26], namely $R_{1}=\frac{1}{2}\log\left(1+\frac{P_{1}}{1+N_{2}+P_{2}}\right)$ and $R_{2}=\frac{1}{2}\log\left(1+P_{2}\right)$. This corresponds to a Phase 3 communication strategy. (At this corner point $R_{2}$ takes its maximum value.)

**Theorem 2**  ([29]). *Consider a Gaussian Z-interference channel. The smallest β such that the supporting hyperplane of the form $R_{1}+\beta R_{2}$ of the Han–Kobayashi signaling scheme with Gaussian inputs passes through the backoff corner point is given by*

$$\beta_{backoff}=1+\max\left\{\frac{N_{2}\left(1+N_{2}+P_{2}\right)}{P_{2}},\frac{\log\left(1+N_{2}\right)-\frac{N_{2}}{\left(1+N_{2}+P_{1}+P_{2}\right)}}{\log\left(\frac{1+P_{2}+N_{2}}{1+N_{2}}\right)-\frac{P_{2}}{1+P_{2}+N_{2}}}\right\}.$$

Thus, for all $\beta \geq \beta_{backoff}$, the supporting hyperplane to the Gaussian signaling region (or the noiseberg region) passes through the above corner point.

**Remark 3.**  *As with the sum-rate point, we do not yet have a matching converse (i.e., one that follows from an outer bound to the capacity region) that establishes the slope at this corner point. An interested reader may refer to [24,27], where upper bounds on the slope have been established. The issue of corner points to the capacity region of two-user Gaussian interference channels in various signal-to-noise ratio regimes has been studied in [30].*

#### 2.2.2. The Intermediate Regime, $\beta :\beta_{Sato}\leq \beta \leq \beta_{backoff}$

The main objective of this paper would be to review the known results for β in the regime $:\beta_{Sato}\leq \beta \leq \beta_{backoff}$. Initially, consider the leftmost (pure superposition coding) strategy, i.e., we only consider Phases 1, 2, and 3. In Phase 2, we need to compute

$$\max_{0\leq P_{1A}\leq P_{1}}\frac{1}{2}\log\left(1+\frac{P_{1}-P_{1A}}{1+N_{2}+P_{2}+P_{1A}}\right)+\frac{1}{2}\log\left(1+P_{1A}\right)+\frac{\beta }{2}\log\left(1+\frac{P_{2}}{1+N_{2}+P_{1A}}\right).$$

A little bit of calculus shows that optimizing

$$\begin{array}{cc} P_{1A} & =\left\{\begin{array}{c} \begin{array}{ccc} P_{1} & \mathrm{if}\;\beta \leq \frac{P_{2}+N_{2}}{P_{2}}\frac{1+P_{1}+N_{2}}{1+P_{1}}, & (\mathrm{Phase}\;1)\\ 0 & \mathrm{if}\;\beta \geq \frac{P_{2}+N_{2}}{P_{2}}\left(1+N_{2}\right), & (\mathrm{Phase}\;3)\\ \frac{\left(1+N_{2}\right)\left(P_{2}+N_{2}\right)-\beta P_{2}}{\beta P_{2}-\left(P_{2}+N_{2}\right)} & \mathrm{otherwise}. & (\mathrm{Phase}\;2) \end{array} \end{array}\right.. \end{array}$$

In the above optimization problem, we observe that there are two transition values for β, defined by $\beta_{1}=\frac{P_{2}+N_{2}}{P_{2}}\frac{1+P_{1}+N_{2}}{1+P_{1}}$ (marking the transition from Phase 1 to Phase 2) and $\beta_{2}=\frac{P_{2}+N_{2}}{P_{2}}\left(1+N_{2}\right)$ (marking the transition from Phase 2 to Phase 3). Note that $\beta_{1}$ corresponds to the first of the two terms in the minimization that defines $\beta_{Sato}$, and $\beta_{2}$ corresponds to the first of the two terms in the maximization that defines $\beta_{backoff}$.

It turns out that the second of the two terms in the minimization that defines $\beta_{Sato}$ corresponds to a phase transition from Phase 1 to Phase 4. Similarly, the second of the two terms in the maximization that defines $\beta_{backoff}$ corresponds to a phase transition from Phase 6 to Phase 3.

Phases 1, 2, and 3 can be considered special instances of Phases 4, 5, and 6, respectively, by setting λ=0. Phase 1 (Sato’s corner point) is associated with the segment λ=0 and $0\leq h\leq N_{2}$. Phase 2 (the pure superposition phase) is related to the middle segment formed by λ=0 and $N_{2}\leq h\leq N_{2}+P_{1}.$ Finally, Phase 3 (the backoff corner point) is mapped to the single point, λ=0, and $h=N_{2}+P_{1}$.

Further, the rate pairs $\left(R_{1},R_{2}\right)$ in Phases 4, 5, and 6 can also be expressed in terms of the parameters λ and *h* as stated before. These parameters λ,h vary over a region, called the region of admissible points, defined by the conditions that $P_{1A}$, $P_{1B}$, and $P_{1C}$ are non-negative and sum to $P_{1}$. The region $h\leq N_{2}$ and λ>0 corresponds to Phase 4. If $h\geq N_{2}$, then $P_{1C}>0$, and this is called the overflow region. This encompasses Phases 5 and 6.

The admissible region in Phase 4, using (1), can be shown to be restricted by the expressions $0\leq h\leq N_{2}$ and

$$0<\lambda \leq \frac{P_{1}+P_{2}+h-\sqrt{{\left(P_{1}+P_{2}+h\right)}^{2}-4P_{1}h}}{2h}.$$

The admissible region in Phase 5, using (3), can be shown to be restricted by the expressions $N_{2}<h\leq N_{2}+P_{1}$ and

$$0<\lambda <\frac{P_{1}+P_{2}+2N_{2}-h-\sqrt{{\left(P_{1}+P_{2}+2N_{2}-h\right)}^{2}-4N_{2}\left(P_{1}+N_{2}-h\right)}}{2N_{2}}.$$

Finally, the admissible region in Phase 6, using (5), can be shown to be restricted by the expressions $N_{2}<h\leq N_{2}+P_{1}$ and

$$\lambda =\frac{P_{1}+P_{2}+2N_{2}-h-\sqrt{{\left(P_{1}+P_{2}+2N_{2}-h\right)}^{2}-4N_{2}\left(P_{1}+N_{2}-h\right)}}{2N_{2}}.$$

**Figure 11:**
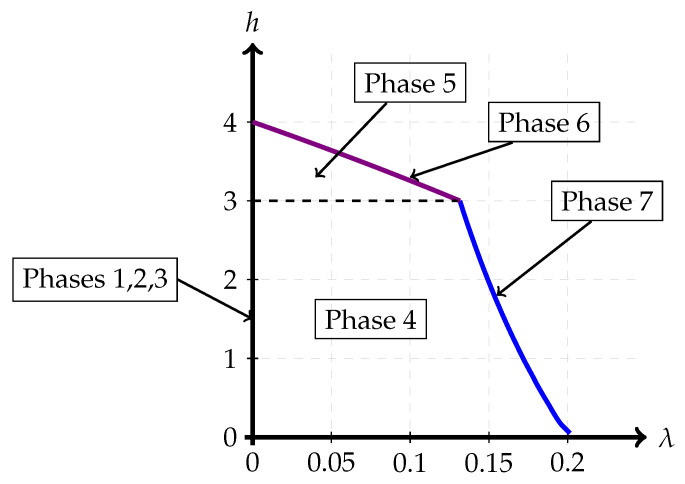
Contour of admissible region for a Z-interference channel with $Q_{1}=1$, $Q_{2}=1$, and a=0.5, i.e., a degraded channel with $P_{1}=1$, $P_{2}=4$, and $N_{2}=3$.

Figure 11 shows the admissible region of λ and *h* for the parameters $P_{1}=1$, $P_{2}=4$, and $N_{2}=3$. Phases 1, 2, and 3 correspond to λ=0 and collapse to the Y-axis. Phase 6 corresponds to the upper boundary. The dotted line at h=3 marks the phase boundary between Phases 4 (noiseberg multiplex) and 5 (overflow). Finally, Phase 7 is the rightmost boundary of the admissible region, which is reached in case the boundary is met before overflow.

To determine the phase, we need to maximize $R_{1}\left(h,\lambda \right)+\beta R_{2}\left(h,\lambda \right)$ (using (2) or (4) depending on $h\leq N_{2}$ or $h\geq N_{2}$, respectively) and this leads to a path of optimal extreme points in the admissible region.

Apart from the phase transitions characterized in Theorems 1 and 2, namely, Phase 1 → Phase 2, Phase 1 → Phase 4, Phase 2 → Phase 3, and Phase 6 → Phase 3, the numerical experiments show that there are five more types of phase transitions in the Gaussian signaling scheme. These other ones represent the transitions from Phase 4 → Phase 5, Phase 4 → Phase 7, Phase 7 → Phase 6, Phase 5 → Phase 2, and Phase 5 → Phase 6, and let us define the corresponding β’s to be $\beta_{4\to 5}$, $\beta_{4\to 7}$, $\beta_{5\to 6}$, $\beta_{5\to 2}$, and $\beta_{5\to 6}$, respectively. These phase transitions can be implicitly characterized as follows:

- $\beta_{4\to 5}$: This corresponds to the β at which $R_{1}\left(h,\lambda \right)+\beta R_{2}\left(h,\lambda \right)$ is maximized when $h=N_{2}$ and λ>0. This corresponds to the transition between the multiplex and overflow regions.
- $\beta_{4\to 7}$: This corresponds to the β at which $R_{1}\left(h,\lambda \right)+\beta R_{2}\left(h,\lambda \right)$ is maximized when $h<N_{2}$ and $\lambda =\frac{P_{1}+P_{2}+h-\sqrt{{\left(P_{1}+P_{2}+h\right)}^{2}-4P_{1}h}}{2h}$, in other words $P_{1A}=0$. This corresponds to the transition from the multiplex region to the non-naïve time-division region.
- $\beta_{7\to 6}$: This corresponds to the β at which $R_{1}\left(h,\lambda \right)+\beta R_{2}\left(h,\lambda \right)$ is maximized when $h=N_{2}$ and $\lambda =\frac{P_{1}+P_{2}+h-\sqrt{{\left(P_{1}+P_{2}+h\right)}^{2}-4P_{1}h}}{2h}$. This corresponds to the transition from the time-division phase to the overflow region, with $P_{1A}=0$.
- $\beta_{5\to 2}$: This corresponds to the β at which $R_{1}\left(h,\lambda \right)+\beta R_{2}\left(h,\lambda \right)$ is maximized when $h>N_{2}$ and λ=0. This corresponds to the transition from the overflow region to a pure superposition coding region.
- $\beta_{5\to 6}$: This corresponds to the β at which $R_{1}\left(h,\lambda \right)+\beta R_{2}\left(h,\lambda \right)$ is maximized when $h>N_{2}$ and $\lambda =\frac{P_{1}+P_{2}+2N_{2}-h-\sqrt{{\left(P_{1}+P_{2}+2N_{2}-h\right)}^{2}-4N_{2}\left(P_{1}+N_{2}-h\right)}}{2N_{2}}.$ This corresponds to the transition from the interior of the admissible (h,λ) region to the top boundary of this region.

#### 2.2.3. Trajectories in the Phase Space

In this section, we plot the various trajectories of the optimizing parameters in the phase space of (h,λ) as we vary β from $\beta_{sato}$ to $\beta_{backoff}$.

**Figure 12:**
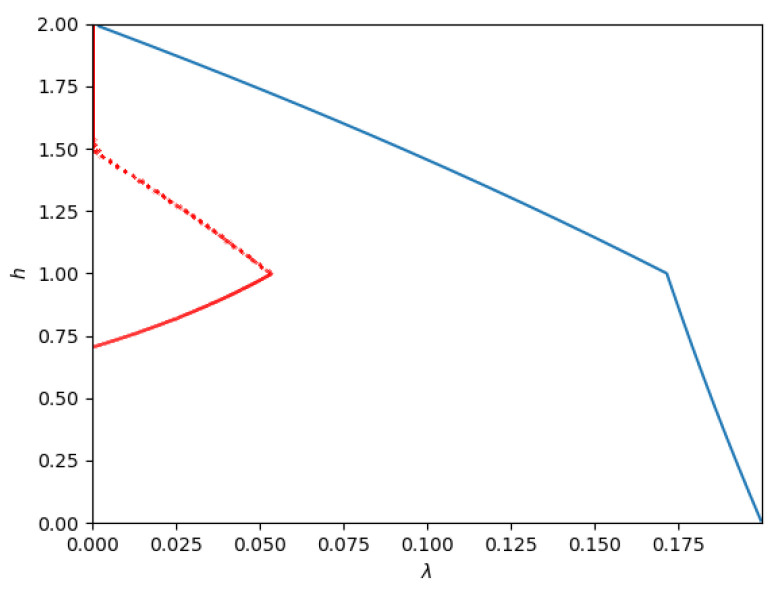
Contourof the admissible region (blue lines) and optimized points (red lines; obtained numerically) for a Z-interference channel with $Q_{1}=1$, $Q_{2}=2$, $a=\frac{\sqrt{2}}{2}$, i.e., a degraded channel with $P_{1}=1$, $P_{2}=4$, $N_{2}=1$.

**Figure 13:**
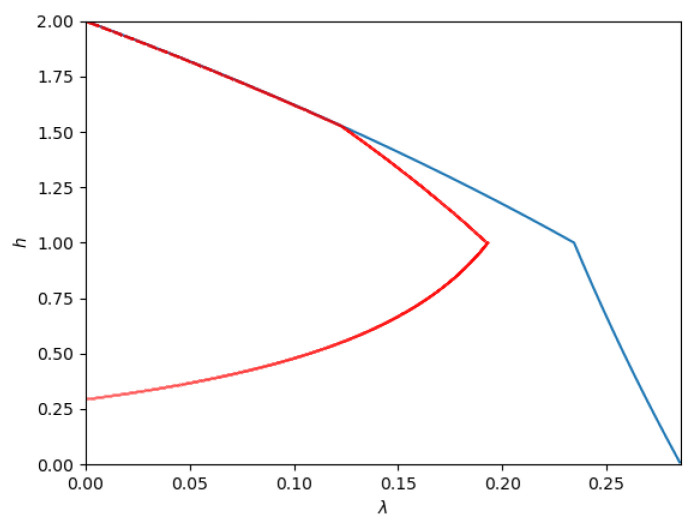
Admissible region (blue lines) and optimized points (red lines; obtained numerically) for a Z-interference channel with $Q_{1}=1$, $Q_{2}=1.25$, and $a=\frac{1}{\sqrt{2}}$, i.e., a degraded channel with $P_{1}=1$, $P_{2}=2.5$, and $N_{2}=1$.

**Figure 14:**
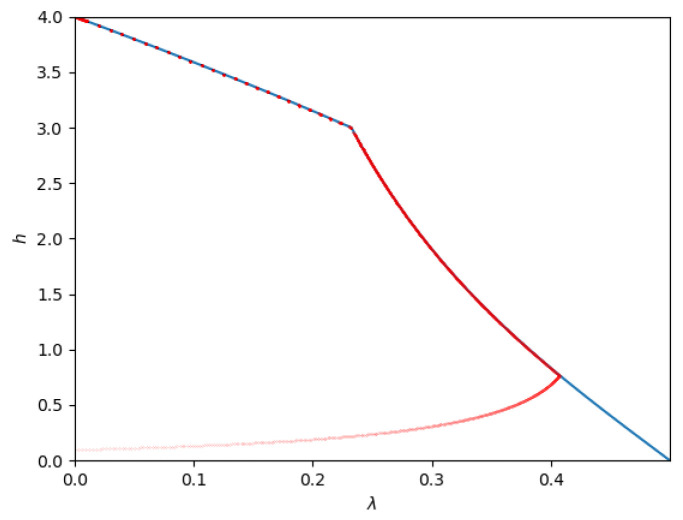
Admissible region (blue lines) and optimized points (red lines; obtained numerically) for a Z-interference channel with $Q_{1}=1$, $Q_{2}=0.25$, and $a=\frac{1}{2}$, i.e., a degraded channel with $P_{1}=1$, $P_{2}=1$, and $N_{2}=3$.

1. **Path 1**: For some set of parameters (for example, $P_{1}=1,P_{2}=9,N_{2}=8$) numerical simulations indicate that the optimal path is Phase 1 → Phase 2 → Phase 3. This is the path of pure superposition evolution, and the locations of the phase transitions occur at $\beta_{1\to 2}=9\frac{4}{9}$ and $\beta_{2\to 3}=17$, respectively. This implies that the trajectory in the admissible region is only along the *h*-axis, i.e., with λ=0.
2. **Path 2**: For some set of parameters (for example, $P_{1}=1,P_{2}=4,N_{2}=1$) the optimal path seems to be Phase 1 → Phase 4 → Phase 5 → Phase 2 → Phase 3. The phase transitions occur at (approximately) $\beta_{1\to 4}=1.8422$, $\beta_{4\to 5}=1.9549$, $\beta_{5\to 2}=2.0799$, and $\beta_{2\to 3}=2.5$, respectively. This path is depicted in Figure 12.
  As the figure illustrates, it leaves Phase 1 (Sato’s point) and moves into Phase 4. Then, at $h=N_{2}=1$, it moves from Phase 4 to Phase 5. Then, at h≈1.5, it moves from Phase 5 to Phase 2. Finally, at h=2, the trajectory reaches the other corner point.
3. **Path 3**: For some set of parameters (for example $P_{1}=1,P_{2}=2.5,N_{2}=1$) the optimal path seems to be Phase 1 → Phase 4 → Phase 5 → Phase 6 → Phase 3. The phase transitions occur at (approximately) $\beta_{1\to 4}=1.9238$, $\beta_{4\to 5}=2.4153$, $\beta_{5\to 6}=2.6987$, and $\beta_{6\to 3}=3.0023$ respectively.
  This path is depicted in Figure 13.
  As the figure illustrates, it leaves Phase 1 (Sato’s point) and moves into Phase 4. Then, at $h=N_{2}=1$, it moves from Phase 4 to Phase 5. At a slightly larger β, it enters Phase 6 and remains there until it reaches the new corner point at Phase 3.
4. **Path 4**: For some set of parameters (for example, $P_{1}=1,P_{2}=1,N_{2}=3$) the optimal path seems to be Phase 1 → Phase 4 → Phase 7 → Phase 6 → Phase 3. The phase transitions occur at (approximately) $\beta_{1\to 4}=5.3182$, $\beta_{4\to 7}=9.5662$, $\beta_{7\to 6}=23.3434$, and $\beta_{6\to 3}=39.2955$, respectively.
  This path is depicted in Figure 14.

### 2.3. To Mux or Not to Mux

From the point of view of a converse to the capacity region, it may be helpful to realize that one does not need multiplexing (time-sharing) for some parameters $\left(Q_{1},Q_{2},a\right)$. Hence, there is a potential for the existing techniques for proving the optimality of Gaussian distributions to work.

In Theorem 1, the authors computed the slope, $\beta_{\mathrm{Sato}}$, of the capacity region at the sum-rate corner point. Whenever the first term there is the critical one, it was observed that the optimal trajectory consists only of the pure superposition phases, i.e., Phases 1, 2, and 3. This leads us to propose the following conjecture.

**Conjecture 1.**  *Consider a degraded Gaussian Z-interference channel with parameters $\left(P_{1},P_{2},N_{2}\right)$. The noiseberg region consists only of a pure superposition coding strategy (i.e., no time-sharing is required for any β-sum-rate) whenever*

$$\frac{\left(N_{2}+P_{2}\right)\left(1+N_{2}+P_{1}\right)}{P_{2}\left(1+P_{1}\right)}\leq \beta^{*},$$

where $\beta^{*}$ is the unique positive solution of ψ(β)=0, where

$$\begin{array}{cc} \psi (\beta )\; & :=\beta \left(\log\left(1+\frac{P_{2}}{1+N_{2}+P_{1}}\right)-\frac{N_{2}P_{2}}{\left(1+N_{2}+P_{1}\right)\left(1+N_{2}+P_{1}+P_{2}\right)}\right)\\ \; & \;+\log\left(1-\frac{P_{2}\left(1+P_{1}\right)}{\left(1+N_{2}+P_{1}\right)\left(1+N_{2}+P_{1}+P_{2}\right)}\beta \right). \end{array}$$

**Figure 15:**
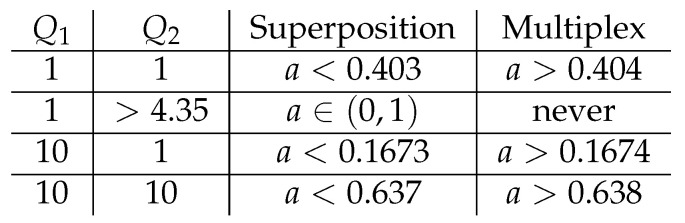
Boundaries between pure superposition and multiplex regions for different values of $Q_{1}$ and $Q_{2}$.

This conjecture allows us to identify the regions of parameters $Q_{1}$, $Q_{2}$, and *a* where we have optimal rate evolution between the extreme points with pure superposition alone, i.e., without the use of noisebergs. The results are shown in Figure 15.

### 2.4. The Other Critical Points

**Figure 16:**
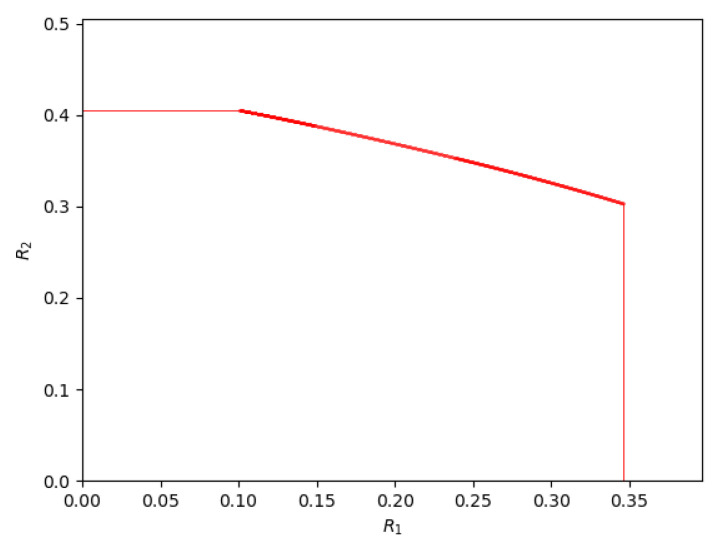
The noiseberg achievable region for $P_{1}=1$, $P_{2}=2.5$, and $N_{2}=1$.

Figure 16 depicts the achievable rate region for the parameters $P_{1}=1,P_{2}=2.5,$ and $N_{2}=1$. Clearly, the rate region depicts a discontinuity in the slope at the sum-rate extreme point as well as the backoff extreme point. Even the capacity region has been shown to exhibit this behavior. However, in an earlier section (cf. Figure 13), we claimed that this set of parameters also exhibits a phase transition at (approximately) $\beta_{4\to 5}=2.415$, where the rates are (approximately) $R_{1}=0.238$, $R_{2}=0.353$ and at (approximately) $\beta_{5\to 6}=2.699$, where the rates are (approximately) $R_{1}=0.149$, $R_{2}=0.388$. The achievable rate region does not exhibit a discontinuity in slope at these parameters. However, these are second-order phase transitions of the capacity region, i.e., the second derivative has a discontinuity. This happens because there is a crossing of the curves of β that correspond to two schemes that are involved in the transition. But β is the slope of the normal to the tangent of the achievable rate curve. Therefore the first derivatives of the achievable rate curve will not show a discontinuity at these critical points, but their second derivatives (the slope of the slope) will.

For the noiseberg region, the phase transitions $\beta_{4\to 7},\beta_{4\to 5},\beta_{7\to 6},\beta_{5\to 6},\beta_{5\to 2}$ all appear to be second-order phase transitions of the capacity region. This is also supported by some back-of-the-envelope calculations, but a formal proof is still absent at this point.

It is rather interesting how the β’s arrange themselves to give an achievable rate region with no kinks other than the two corner points. For a general scalar Gaussian interference channel, in a very weak-interference regime, it is known that the capacity region exhibits a kink [31,32] at the maximum sum-rate point as well. In the Z-interference channel case, the sum-rate point coincides with one of the corner points, perhaps indicating that there are no additional kinks for a general scalar Gaussian interference channel.

## 3. Conclusions

In this paper, we review new critical points in the noiseberg achievable rate region of the Gaussian Z-interference channel. This region (and the capacity region) has been known to have two critical points at its two corner points. The noiseberg achievable region is observed to have additional points of second-order phase transitions. One of these critical points ($\beta_{4\to 5}$) marks the transition of the noiseberg multiplex region into the overflow region. Another of these ($\beta_{4\to 7}$) marks the transition of the noiseberg multiplex region into the time-division region. A third critical point ($\beta_{5\to 2}$) marks the transition of the overflow region back to the superposition coding region. Finally, a fourth critical point ($\beta_{5\to 6}$) marks the transition of the overflow region into a phase where the weaker receiver decodes the interfering signal in its band (Phase 6).

It is hoped that the review of the noiseberg region will help the community resolve the following key open-question: does the noiseberg region coincide with the capacity region of the Gaussian Z-interference channel?

## Data Availability

The raw data supporting the conclusions of this article will be made available by the authors on request.
